# Toxemia in Human Naturally Acquired Botulism

**DOI:** 10.3390/toxins12110716

**Published:** 2020-11-13

**Authors:** Christine Rasetti-Escargueil, Emmanuel Lemichez, Michel R. Popoff

**Affiliations:** Unité des Toxines Bactériennes, UMR CNRS 2001, Institut Pasteur, 75015 Paris, France; christine.rasetti-escargueil@pasteur.fr (C.R.-E.); emmanuel.lemichez@pasteur.fr (E.L.)

**Keywords:** botulism, botulinum neurotoxin, *Clostridium botulinum*, toxemia, serum

## Abstract

Human botulism is a severe disease characterized by flaccid paralysis and inhibition of certain gland secretions, notably salivary secretions, caused by inhibition of neurotransmitter release. Naturally acquired botulism occurs in three main forms: food-borne botulism by ingestion of preformed botulinum neurotoxin (BoNT) in food, botulism by intestinal colonization (infant botulism and intestinal toxemia botulism in infants above one year and adults), and wound botulism. A rapid laboratory confirmation of botulism is required for the appropriate management of patients. Detection of BoNT in the patient’s sera is the most direct way to address the diagnosis of botulism. Based on previous published reports, botulinum toxemia was identified in about 70% of food-borne and wound botulism cases, and only in about 28% of infant botulism cases, in which the diagnosis is mainly confirmed from stool sample investigation. The presence of BoNT in serum depends on the BoNT amount ingested with contaminated food or produced locally in the intestine or wound, and the timeframe between serum sampling and disease onset. BoNT levels in patient’s sera are most frequently low, requiring a highly sensitive method of detection. Mouse bioassay is still the most used method of botulism identification from serum samples. However, in vitro methods based on BoNT endopeptidase activity with detection by mass spectrometry or immunoassay have been developed and depending on BoNT type, are more sensitive than the mouse bioassay. These new assays show high specificity for individual BoNT types and allow more accurate differentiation between positive toxin sera from botulism and autoimmune neuropathy patients.

## 1. Introduction

Botulism is a neurological disease of man and animals which is characterized by flaccid paralysis and inhibition of secretions from glands controled by cholinergic innervation, such as salivary, lachrymal, and sweat glands. The flaccid paralysis leading to respiratory distress is responsible for death in the most severe cases. Botulism results from the blockade of neurotransmitter release, notably acetylcholine at the neuromuscular junctions and other cholinergic neurons, by potent neurotoxins called botulinum neurotoxins (BoNTs), which proteolytically cleave SNARE (soluble N-ethylmaleimide-sensitive-factor attachment protein receptor) proteins involved in the exocytosis of neurotransmitters [1,2,3]. BoNTs are synthesized by *Clostridium* species named *Clostridium botulinum* and more rarely by other *Clostridium* species such as toxigenic strains of *Clostridium baratii*, *Clostridium butyricum*, and *Clostridium argentinensis*. According to their immunological properties based on neutralization by specific antisera, BoNTs are currently divided into 9 toxinotypes (A, B, C, D, E, F, G, H, or H/A, or F/A, X) [1,4]. BoNT amino acid sequences deduced from bont gene sequencing revealed that each toxinotype is subdivided into an increasing number of subtypes defined as >2.6% amino acid variation. Thus, 41 BoNT subtypes and multiple variants have been reported [5,6,7]. More recently, genome comparison showed the presence of *bont* related sequences in a few non-clostridial strains such as *bont*/Wo or *bont*/I from *Weisenella oryzae*, a bacterium of fermented rice, *bont*/J (*ebont*/F or *bont*/En) from an *Enterococcus faecalis* strain in a cow, and Cp1 from *Chryseobacterium piperi* from sediment [1,8,9,10,11,12]. However, no active BoNT responsible for human or animal botulism has been reported in the non-clostridial strains. In addition, a BoNT-like neurotoxin (PMP1) specific of invertebrate (*Anopheles* mosquito) has been characterized from a *Paraclostridium bifermentans* strain [13]. Albeit all BoNTs induce the same toxicological effects resulting in flaccid paralysis, most BoNT types and subtypes share a similar mechanism of action but differ by interaction with distinct receptors and intracellular SNARE targets cutting at different cleavage sites [2,14,15].

According to BoNT acquisition, different forms of human botulism are described. Ingestion of preformed BoNT in food is responsible for food-borne botulism, which is the most common form of botulism in many countries. Foods which are non-acidified, no or minimally heated, and contaminated by *C. botulinum* spores, are at risk to allow the growth of *C. botulinum* and BoNT production during their storage even at low temperature [16,17]. Ingested *C. botulinum* spores might lead to a toxico-infection under certain circumstances by colonization of the intestinal tract and in situ BoNT production. Children under the age of 1 year are susceptible to intestinal colonization by *C. botulinum* and to develop infant botulism [18,19]. Perturbations or limited functionality of the developing and yet immature infant gut microbiota are considered as a main risk factor [20,21]. More rarely, botulism by intestinal colonization (also called intestinal toxemia botulism) occurs in children above 1 year and adults. Factors that might impair the regular intestinal microflora such as bowel surgery, bowel anomalies, antibiotics, chemotherapy, radiation, immunosuppressive drugs, and altered nutritional patterns have been reported to be predisposing factors for adult intestinal toxemia botulism [22]. Wound botulism results from a wound contaminated with *C. botulinum* spores and subsequent in situ growth of *C. botulinum* and BoNT production. Moreover, aerosolized BoNT induces rare cases of laboratory botulism [23]. Iatrogenic botulism has been reported with toxin overdoses for therapeutic or cosmetic use [24,25,26,27].

The paradigm of foodborne botulism is that the ingested BoNT, which is preformed in contaminated food, transits through the digestive tract, crosses the intestinal barrier, reaches the blood and lymphatic circulation and disseminates to the peripheral nervous system [28]. In in vitro culture or in contaminated food, BoNT is produced in a complex form (also called progenitor toxin) by association with non-toxic proteins, including notably the non-toxic non-hemagglutinin (NTNH) and hemagglutinin (HA) proteins [25,29,30,31]. A main role of the associated proteins is to prevent BoNT degradation from the stomach acidic pH and digestive proteases. Indeed, NTNH, which shares a similar structure with BoNT, forms an interlocked complex with BoNT which is highly resistant to low pH and protease inactivation [32,33]. The precise mechanism of entry through the intestinal barrier of BoNT and/or the whole BoNT complex is still unclear. Free BoNT is able to pass through intestinal epithelial cells by transcytosis. In addition, HAs have been found to bind to intestinal cells, to disrupt the E-cadherin intercellular junctions between enterocytes, and thus to facilitate the paracellular passage of BoNT complexes. However, according to the distinct *C. botulinum* toxinotypes, numerous BoNT complexes lack HAs but contain other non-toxic proteins (OrfX1, OrfX2, OrfX3, P47) that have not been observed to be involved in the uptake of BoNT from the digestive tract [28,34,35,36]. Therefore, subsequently to the absorption from the intestinal tract, the passage of BoNT in the blood circulation seems to be a hallmark of BoNT intoxication [2,28]. Thus, the demonstration of BoNT in biological samples such as serum allows the confirmation of clinical botulism [24].

Treatment of botulism is based on supportive care, including mechanical ventilation in severe cases and botulinum antiserum. Equine anti-botulinum sera are available for adult botulism, and human anti-BoNT/A and -BoNT/B immunoglobulins (BabyBIG^®^) have been developed for infant botulism [24,37,38,39,40]. Anti-BoNT antibodies are the only specific treatment of botulism. However, since anti-BoNT antibodies mainly neutralize the toxins prior to their binding to neuronal cells, serotherapy’s efficacy depends on the rapid administration of antibodies after the onset of symptoms, ideally <24 h [24]. Therefore, early diagnosis of botulism is critical for the management of botulism. Botulism cases are suspected on the basis of clinical symptoms and epidemiological aspects. Botulism confirmation is supported by laboratory tests that are required for botulism typing [41,42]. Initially, food-borne botulism was confirmed by BoNT detection in suspected food samples using experimental animals [40]. Thus, from 1940 to 1970 the diagnosis of botulism in France at Institut Pasteur was performed only by BoNT investigation in food [43]. However, it was observed that BoNT can be identified in the serum or blood samples from patients with clinical botulism. Before 1970, some publications reported the presence of BoNT in the sera of patients with botulism type B or E and more rarely type A. From 1905 to 1962, 12 reports demonstrated BoNT in the sera of 20 human cases of botulism up to 25 days after the ingestion of contaminated food (reviewed in [44]). Two additional observations in 1963 and 1965 showed toxin positive sera in 5 and 4 patients with botulism type E and B, respectively [44,45]. From 1970, the detection of BoNT in serum samples was more routinely used for the diagnosis of botulism. Since the BoNT levels that have been observed in human sera are generally low, a high sensitive method of BoNT detection is required. The mouse bioassay which is the standard procedure and allows the detection of a few pg of BoNT per mL, was used in almost all studies. BoNT typing is performed by neutralization with specific anti-BoNT type antibodies [42]. This review focusses on BoNT detection in sera from patients with naturally acquired botulism. Since BoNT detection in serum is the most direct way to confirm a diagnostic of botulism, it is important to know the parameters of botulinum toxaemia such as prevalence of toxaemia according to botulism forms, duration, serum toxin levels, in order to adapt the serum sampling according to the onset of the disease and to interpret possible false negative/positive results.

## 2. Detection of BoNT in the Sera of Patients with Food-Borne Botulism

Investigations of BoNT detection in the sera of patients with food-borne botulism have been reported mainly since the 1970s (Table 1). Overall, from 1456 reported testings of BoNT by the mouse bioassay in the sera of patients suspected of botulism, 633 (43%) were positive (Table 1). However, a high heterogeneity is observed between the different investigations. Some of them concerned a small number of case reports or small series of patients (1 to 50) with a high incidence of positivity (100 to 56%). In other larger investigations, the detection of BoNT in sera was lower (about 20 to 35%). The clinical status of the patients who have been tested was not fully defined in all the reports. The detection of BoNT in the serum of patients with clinical symptoms of food-borne botulism is around 70% (56–82%) whatever the BoNT type, as indicated in the most documented publications [46,47,48,49,50,51]. BoNT is exceptionally reported in the serum of asymptomatic persons who ate the same contaminated foods as patients from a same outbreak of botulism. For example, BoNT/B was detected in the serum of two asymptomatic persons 87 days after ingestion of contaminated ham [43]. It was also reported BoNT/E and possibly BoNT/A in the serum of two asymptomatic cases 7 and 8 days, respectively, after consumption of toxic foods [44,45].

BoNT level in the patient’s sera depends on the ingested amount of toxin and the time elapsed between the ingestion of toxin and serum collection. In addition, variations of serum BoNT levels between patients from the same botulism outbreak have been observed. These individual variations likely result from different abilities in intestinal absorption of BoNT and/or clearance/elimination of the toxin, the mechanisms of which remain to be defined [28].

The levels of BoNT in naturally contaminated foods are very variable from <10 mouse lethal doses (MLD)/g to 280,000 MLD/g, as reported in the following publications [47,48,49,68,70,82]. The amount of ingested toxin by each patient can be estimated from the approximate portion which has been consumed, but it is usually unknown. Individual variations in the susceptibility to botulism are illustrated by the following example. An outbreak of botulism occurred in 2010 in Corsica in a family of 7 persons who ate a salad with home-canned green beans containing 2000 MLD/g BoNT/A2. According to the hostess, each person consumed an equivalent portion of salad. An 18-year-old patient developed a severe form of botulism and died of sudden respiratory arrest in less than 24 h. Three persons presented severe botulism and required mechanical ventilation for 37 to 78 days. Another guest showed a moderate form of botulism (diplopia, dysphagia, dysarthria), and the two remaining persons were asymptomatic. The levels of BoNT/A in the serum samples collected 1–2 days after the contaminated meal were 8 MLD/mL from the patient who died, 4 MLD/mL from two and 32–64 MLD/mL from one of the three patients who required intensive care, 4 MLD/mL from the patient with moderate botulism, and under the limit of detection from the two asymptomatic persons [49,83]. It is noteworthy that no obvious correlation was observed between the serum BoNT levels and the severity of symptoms [47]. However, high BoNT concentration in serum, notably BoNT/A, shortly (1–4 days) after ingestion of contaminated food is frequently associated with a severe form of botulism.

The levels of BoNT in the serum of patients are usually low. Most of the reported serum BoNT levels (>99%) range from 1 to 10 MLD/mL (Table 2). In some cases, the BoNT levels are <1 MLD/mL. Thus, injection of 1 mL of the patient’s serum into mice induces characteristic paralysis symptoms without lethality, which are neutralized by specific anti-BoNT antibodies. A small number of patients showed higher BoNT levels from 16 to 40 MLD/mL. Exceptional very high serum BoNT levels were reported (Table 2) such as 1800 MLD/mL of BoNT/A in a patient intoxicated with commercial carrot juice [68], and 400 MLD/mL of BoNT/F in a food-borne botulism case from an unknown origin [84,85].

The timeframe between ingestion of toxin and serum sampling is highly critical for successful BoNT detection. Indeed, the chances of obtaining positive results decrease when the time between the onset of symptoms and serum sampling increases. Most of the reported timeframes with positive BoNT detection in patient’s sera ranged from 1 to 4 days, whatever the botulism type (Table 3). In a series of 340 patient’s sera, BoNT was detected in >60% of serum samples collected within the two days after toxin ingestion, and positive results dropped to 13–28% in the sera obtained later [51]. However, long-lasting toxemia can be observed in certain patients. Most of the prolonged toxemia (more than 10 days) were observed in botulism type B (Table 2 and Table 3). Thereby, from 28 cases of botulism type B, the toxin was detected 2–3 days post-intoxication in the sera of 3 patients, 6 to 21 days in 23 patients, and 87 and 122 days in 2 other persons [43]. Sheth et al. [68] reported the presence of BoNT/A in the sera of two patients 12 and 25 days after illness onset. In another investigation, including 64 toxin positive sera, toxemia was detected in 15 patients 4 to 11 days after toxin ingestion [55] (Table 3). In an outbreak of botulism type A, the toxin was identified in the sera of 3 of 9 patients 10–12 days after toxin exposition [59]. Toxin detection in sera from patients with botulism type E or F was reported no more than 8 days after the disease onset (Table 2 and Table 3). Therefore, the chances of toxin detection in patient’s sera are maximal when serum sampling is as early as possible after toxin ingestion (ideally 1 to 4 days), but in some cases, very prolonged toxemia up to more than 120 days can be observed. The individual variations in the duration of botulism toxemia remain to be determined. L. Simpson suggests that BoNT elimination from the blood circulation most likely is achieved through hepatic uptake and biotransformation. The very low toxin levels in the blood, which are frequent in botulism type B, could not to be detected by the elimination system, thus accounting for prolonged toxemia [28].

## 3. Detection of BoNT in Sera of Patients with Intestinal Colonization

### 3.1. Infant Botulism

Botulism by intestinal colonization is characterized by the intestinal local production of BoNT, which is able to cross the intestinal barrier and to directly target the intestinal innervation. Efficient specific therapy for infant botulism consists of human botulism immunoglobulins (BabyBIG, BIG-IV) [39,86]. Experimentally, BoNT/A and BoNT/B administrated into the lumen of mouse intestinal loop crossed the epithelial cell barrier and specifically recognized neurons, mainly cholinergic neurons, in the intestinal submucosa and musculosa within 20–60 min [34,87,88]. Indeed, in addition to progressive weakness, a typical early symptom of infant botulism is constipation [18,19,21,89]. This suggests that synthesized BoNT in the intestine is, in part, eliminated with the stools, and another part is directly trapped by the enteric nervous system resulting in loss of intestinal motility and secretion. Thus, no remaining toxin or only a small fraction of remaining toxin is available for transport to the blood and lymph circulation. In contrast, in food-borne botulism, the ingestion of a BoNT bolus, likely in a larger amount than the toxin synthesized locally in the intestine, leads to a more abundant toxin proportion being absorbed and transported into the general circulation. Thereby, the presence of BoNT in the sera of infants with botulism has been less frequently reported than in sera from patients with food-borne botulism. Among 112 investigated serum samples from infant botulism, about 28% were toxin positive (Table 4), compared to the estimated 70% positivity in food-borne botulism (see above). In the large series of suspected infant botulism in the US, BoNT was identified in 9 sera of 67 infants (13%) with positive *C. botulinum* culture feces and none in 114 sera from infants with negative culture feces. In contrast, the toxin was recovered in the stools of most (87%) of the culture-positive infants [90]. Thus, the presence of neurotoxigenic *Clostridium* and toxin in feces seems to be the hallmark of infant botulism, while toxemia is less frequent. Therefore, laboratory confirmation of infant botulism mainly results from the detection of *C. botulinum* and BoNT in stool samples.

Most of the toxin positive sera from infant botulism are observed within 3–4 days after the symptom onset (Table 5), albeit neurotoxigenic *Clostridium* and toxin may be present in stools for up to several months [89,103,107,112,113,114]. In only a few cases, toxemia was recorded within a longer period, 7 to 27 days [92,93,113]. The toxin concentration in the sera from infant botulism is low (1 to 8 MLD/mL) and often at the limit of detection by the mouse bioassay [48,49,93], despite high BoNT levels in the intestinal content. For example, BoNT was not detected in the sera from 4 infants whose stools contained BoNT ranging from 2000 to 300,000 MLD/g [48,107]. This further argues for the weak transport efficiency of BoNT through the intestinal barrier and subsequent passage in the blood circulation [28,87] and supports that the largest part of toxin produced in the intestine during the clostridial growth is likely eliminated with the stools.

### 3.2. Botulism by Intestinal Colonization

More rarely, botulism by intestinal colonization occurs in infants above 1 year and in adults (adult intestinal toxemia botulism). Predisposing factors allowing spore germination, bacterial growth, and toxin production in the intestine include recent use of antibiotics, which can perturb the resident microbiota, previous gastric and/or intestinal surgery, and bowel anomalies such as Meckel’s diverticulum [22,95,115]. In a limited number of cases, the pathophysiology of botulism by intestinal colonization is similar to that of infant botulism. Of 12 cases reported in the literature with toxin investigation in the serum, BoNT was identified in 10 of them (Table 6). Most sera (9/12) were toxin positive when collected between 5 and 22 days after symptom onset. The longest timeframe was observed in a 27–year old man with botulism type B 47 days after the ingestion of suspected food. The onset of symptoms coincided with the appearance of toxin in the serum, suggesting a progressive *C. botulinum* growth and toxin production in the intestine [116].

## 4. Detection of BoNT in the Sera from Patients with Wound Botulism

Wound botulism is prevalent in certain countries such as California and UK (Table 7), and is mainly associated with risk factors, notably drug injection and more rarely accidental or surgically infected wounds. Local growth of *C. botulinum* in the anaerobic condition of infected wound leads to production of toxin, which diffuses through the extracellular space of the tissues to the local nerve endings and to the blood circulation. It is noteworthy that the clinical signs are identical to those of food-borne botulism but without gastrointestinal symptoms [24]. Of 335 patients with wound botulism reported in the literature, BoNT was identified in about 70% of them (Table 7). Toxin detection in the serum of patients with wound botulism is similar to that in food-borne botulism cases and higher than that in botulism by intestinal colonization. The time of collection of serum samples has not been reported in the cases of wound botulism. We can assume that serum sampling has been done shortly after the onset of symptoms corresponding to the maximum chances of toxin recovery in serum. The incubation period of wound botulism is estimated to 2–18 days [125,126]. In patients with late-onset of symptoms, 7–18 days after the initial wound contamination, a slow and progressive release of toxin with low amount in the blood circulation under the detection limit might account for the negative BoNT detection in serum.

## 5. Detection of Toxin in Sera of Patients with Neuropathic Symptoms Mimicking Botulism

Descending flaccid paralysis of botulism might be confused with neuropathic syndromes such as Guillain-Barré (GBS), Miller Fischer (MFS), Eaton-Lambert syndrome (ELS) or myasthenia [24]. Notably, some atypical cases of these neuropathies or myasthenia are difficult to distinguish clinically from botulism. GBS and MFS frequently mimic botulism [24,135,136,137]. GBS and MFS are immune neuropathies characterized by high titers of serum antibodies against gangliosides, in particular anti-GM1, anti-GD1a, anti-GD1b, anti-GT1b in GBS, and anti-GQ1b in MFS [138,139,140,141,142,143]. The laboratory confirmation of the differential diagnosis between botulism and autoimmune neuropathies is based on the identification of BoNT or antiganglioside antibodies in the patient’s sera, respectively. However, mouse bioassay can detect a toxicity that is not related to BoNT and which might lead to a false diagnostic of botulism. Indeed, toxicity of the patient’s serum by the mouse bioassay has been found in some cases of GBS and MFS (Table 8). Thus, intraperitoneal injection of serum from certain GBS patients into mouse induce respiratory distress and muscle paralysis similarly to BoNT administration [144]. Most often, the toxicity of serum from patients with GBS or MFS is not specifically neutralized by anti-botulinum toxin type antibodies leading to a conclusion of serum toxicity not related to BoNT [47,144,145]. In rare cases, BoNT/B or BoNT/A has been falsely identified in sera from MFS patients, but the presence of high level of anti-GQ1b antibodies or electromyography investigation supported the diagnosis of MFS [146,147]. The identification of BoNT toxinotype is done when only the mice injected with the patient’s serum sample incubated with the corresponding anti-BoNT toxinotype survive and all other mice die. However, if the patient’s serum’s toxicity is low ((1 LD50/mL), some mice can survive, leading to a false neutralization by a specific antiserum. It was observed that additional serum samples 5–6 days after a first toxin positive serum from GBS patients were no longer toxic [145]. Thus, serum toxicity from GBS and MFS patients seems to be low and for a short duration. The toxicity of the serum from patients with autoimmune neuropathy detected by the mouse bioassay is likely due to the autoantibodies against gangliosides. Indeed, antibodies against the gangliosides GM1, GD1a, GQ1b, or acetylcholine receptor impair acetylcholine release at neuromuscular junctions and mediate injury of motoneurons leading to muscle paralysis in mice [148,149,150,151,152,153,154]. Moreover, anti-GM1 antibodies induce flaccid paralysis in rabbit [155]. Monoclonal antibodies against GM1 and GD1a reduce the neurotransmitter release by decreasing the depolarization-induced calcium influx [156]. Anti-GQ1b and anti-GT1a block the transmission at neuromuscular junctions in a complement-dependent manner [151,157]. It is noteworthy that the gangliosides GD1a, GT1b, GQ1b are neuronal membrane targets of both BoNTs and autoantibodies [3,158], that might account for the similarity of symptoms between botulism and autoimmune neuropathies.

## 6. Alternative Methods of BoNT Detection in Serum

Since, in most cases, the BoNT level in the patient’s sera is low, a highly sensitive and specific method of toxin detection is required. The mouse bioassay is very sensitive, but it needs a laboratory with facility and authorization for animal handling. In addition, this in vivo assay might take up to 24–96 h, and it might interfere with non-related BoNT toxicity of sera from autoimmune neuropathies. Alternative in vitro methods of BoNT detection are based on immunological detection such as ELISA or other immunoassays, as well as on the BoNT enzymatic activity towards their specific SNARE protein substrates (SNAP25, VAMP, syntaxin) (Table 9) [42,161,162,163,164]. The sensitivity of the in vitro methods that have been developed with spiked or clinical human sera, is similar or lower than that of the mouse bioassay. Higher sensitivity than the mouse bioassay has been reported for some BoNT types, notably A and B (Table 8). However, the in vitro methods do not detect the full biological activity of BoNTs. Most of the in vitro methods use a two-step strategy, including a BoNT immunocapture with specific polyclonal or monoclonal antibodies most often directed against the Hc domain, followed by an endopeptidase assay with optimized SNARE peptide substrates. The cleavage products of the SNARE peptides are monitored either by immunological, fluorimetric, or mass spectrometry (MS) methods (Table 8). Immunological detection of the SNARE cleavage products benefits from the generation of neoepitopes in BoNT cleaved SNARE proteins. Thereby, specific antibodies recognize SNARE substrates cleaved by each BoNT type and not the native SNARE proteins (reviewed in [161,165]). The advantages of the in vitro methods are the rapidity (2.5 to 6 h) and the precise determination of the BoNT type, thus allowing accurate differentiation between botulism and autoimmune neuropathies as tested with clinical human sera [166,167] (Table 8). However, the in vitro assays require specific development for each BoNT type, while the mouse bioassay is sensitive to all BoNT types, and immunoassay detection is not specific of BoNT active forms.

## 7. Concluding Remarks

The paradigm of the physiopathology of botulism consists of BoNT absorption from the digestive tract/respiratory tract/wound, passage into the blood circulation, and then distribution to the target neurons [2,28]. The pharmacokinetics of BoNT has been investigated in experimental animals (mouse, rat) after intravenous or oral toxin administration. Orally administrated BoNT is detectable in the blood circulation after a latency period (5 h in mice receiving 1 or 4 μg BoNT/A1 complex by oral route) [169]. BoNT that reaches the blood circulation shows a biphasic decline: an initial and short phase with the maximum levels of BoNT, then a long phase with progressive BoNT decrease. The first phase is considered as the distribution phase of BoNT to the extracellular fluid compartments and the second slow phase as the elimination phase and clearance of BoNT from the blood circulation. BoNT is essentially free in the serum/plasma without binding to blood cells, only a small fraction (24%) of BoNT is bound to albumin. BoNT remains highly stable in the blood circulation. No proteolytic degradation, structural change, or loss of activity was observed. Thus, the blood circulation is considered as a “holding compartment”. BoNT elimination from the blood likely results from liver uptake and hepatic biotransformation [28,169,187,188,189]. BoNT in blood has been reported to be free toxin molecules (150,000 Da) non-associated to non-toxic molecules of botulinum complexes [119,190]. It is not clear whether the whole botulinum complexes enter the blood circulation and then dissociate or whether only free BoNT molecules have access to the blood compartment.

The pharmacokinetics of BoNT in humans has not been fully defined. It probably shows a similar pattern than in experimental animals, notably supporting the long persistence of BoNT in the serum of certain patients. BoNT detection in serum is the most direct way to confirm a diagnosis of botulism [41,42,191]. However, the presence of BoNT in the blood circulation depends on the time between serum sampling and the symptom onset, the initial amount of toxin involved in the disease, which has been ingested or produced locally, and probably of individual host factors related to toxin absorption and/or elimination which remain to be defined. BoNT levels in the patient’s sera is most commonly very low, thus requiring highly sensitive and specific methods of detection. In most cases, positive BoNT detection in serum is obtained when the serum sampling is performed within a few days after the disease onset. Based on the reported investigations, BoNT was detected in the serum of about 70% of food-borne botulism and wound botulism patients, and only in 28% of infant botulism. The number of investigations in older infants and adults with botulism by intestinal colonization was too small to be significant. BoNT produced locally in the intestine is likely eliminated in most part by the digestive tract. Only a BoNT fraction probably acts locally, leading to constipation that is a frequent early symptom of infant botulism, and a further smaller fraction enters the blood circulation. In contrast, the whole BoNT amount produced in a contaminated wound is presumed to be available for local activity and dissemination to the blood circulation, accounting for the higher prevalence of toxemia in wound botulism than in infant botulism.

Whether the mouse bioassay is still the most sensitive routine laboratory method to confirm clinical botulism by BoNT detection in serum, alternative in vitro methods such as based on the endopeptidase activity are promising. Their sensitivity is higher than that of the mouse bioassay for certain BoNT types, thus improving the diagnosis of botulism. The in vitro methods offer the advantage to identify more accurately the BoNT types and thus to better differentiate botulism from the autoimmune neuropathies and myasthenia.

## Figures and Tables

**Table 1:** Detection of BoNT in the sera of patients with food-borne botulism.

Country	Year	Number of Patient’s Sera	Toxin-Positive Sera ^1^	BoNT Type					References
				A	B	E	F	Non-Identified	
USA	1905–1962	20	20	1	9	3		7	[44]
USA	1963	6	5			5			[44]
USA	1965	4	4		4				[45]
France	1970–1973	39	28		28				[43]
USA	1975–1988	340	126	71/109	12/109	26/109			[51]
South Africa	1977	2	1	1					[52]
USA	1978	18	11	11					[53]
Japan	1984	1	1		1				[54]
France	1966–1990	50	28		28				[50]
Alaska	1959–2007	180	64	1/15 ^2^	11/15 ^2^	3/15 ^2^			[55]
USA	1991	1	1				1 ^3^		[56]
USA	1993	5	2	2					[57]
Finland	1997	1	1			1			[58]
Argentina	1998	9	3	3					[59]
Finland	1999	1	1			1			[60]
Morocco	1999	11	6		6				[61]
Poland	1990–2000	32	24		23	1			[62]
Canada	1985–2005	212	74	(8%) ^4^	(6%) ^4^	(86%) ^4^			[63]
USA	2001	1	1				1 ^3^		[64]
Germany	2003	1	0 ^5^						[65]
USA	2005	1	1				1 ^3^		[66]
Turkey	2005	9	2	2					[67]
USA	2006	6	5	5					[68]
Finland	2006	1	1			1			[69]
USA	2007	8	2	2					[70]
USA	2007	2	2				2 ^3^		[71]
USA	2007	5	0 ^6^						[72]
Brazil	2000–2008	45	17	8	4 ^7^			5	[73]
France	2007–2009	34	32	5	26	1			[47]
France	2010–2012	51	41	20	18	2		1	[49]
Finland	2011	2	2		2				[74]
UK	2011	2	2	2					[75]
Japan	2012	2	2	2					[76]
Poland	2013	1	1		1				[77]
USA	2015	19	19	19					[78]
Italy	2015	1	1		1				[79]
Italy	1986–2015	275	56	11	45				[80]
France	2013–2016	56	44	7	33		4 ^3^		[48]
Germany	2018	2	2	2					[81]
Total		1456	633						

^1^ Toxin detection in patient’s sera by mouse bioassay, ^2^ BoNT typing was reported for only 15 serum samples, ^3^ *C. baratii* producing BoNT/F detected in stool or food, ^4^ Distribution of the botulism types during this period, ^5^ BoNT/B detected in remaining ham, ^6^ BoNT/E detected in remnant salted fish, ^7^ Detection of BoNT types A and B.

**Table 2:** BoNT concentration (mouse lethal dose (MLD)/mL) in the sera of patients with food-borne botulism.

Country	Year	Number of Patient’s Sera	Days from Symptom Onset to Serum Sampling	BoNT Type	BoNT Concentration (MLD/mL)	References
USA	1963	5	1–10	E	1–10	[44]
France ^1^	1970–1973	1	120	B	1	[43]
		17	3–20 (most 8–15)	B	1–10	
		1	21	B	4–40	
Japan	1984	1	4	B	1–2	[54]
Finland	1997	1		E	6	[58]
Finland	1999	1	1	E	1	[60]
Turkey	2005	9		A	<1–2	[67]
USA	2006	1 (patient A)	1	A	>20	[68]
		1 (patient B)	5	A	>200	
			7		1800	
			8		>200	
			12		>200	
France	2007–2016	1		A	80	[47,48,49]
		2		A	32–40	
		2		A	8–16	
		35		A	1–8	
		6		B	16–40	
		67		B	1–8	
		14		B	<1	
		1		E	8	
		2		E	1	
Finland	2011	1	3	B	<1	[74]
France	2014	1	2	F (*C. baratii*)	400	[84,85]
		1	2		1–2	
France	2015	3		F (*C. baratii*)	1-4	[85]

^1^ BoNT concentration determined in 19 sera of 28 toxin positive sera.

**Table 3:** Detection of botulinum toxin in sera of patients with food-borne botulism and time between the symptom onset and serum sampling.

Country	Year	Days from Symptom Onset to Serum Sampling	Toxin Positive-Sera	BoNT Type	Additional Detection	References
France	1970–1973 ^1^	2	2	B		[43]
		6–18	16			
		20–21	2			
		87	2			
		120	1			
USA	1975–1988	0–3	60–70% ^2^	A, B, E		[51]
		≥ 4	13–28% ^2^			
Japan	1984	4	1	B		[54]
Alaska	1959–2007	4–11	11	B		[55]
		5–7	3	E		
		4	1	A		
USA	1991	4	1	F ^3^	neg at day 14	[56]
Argentina	1998	10–12	3	A		[59]
Finland	1999	1	1	E		[60]
USA	2001	2	1	F ^3^	neg at day 6	[64]
USA	2005	1 and 8	1	F ^3^		[66]
USA	2006	1	3	A		[68]
		5, 7, 8, 12	1 (patient B)	A		
		25	1	A	neg at day 41	
Finland	2006	1	1	E		[69]
USA	2007	3	2 ^4^	A		[70]
		4, 9, 18, 25	neg 6 ^4^			
USA	2007	3, 6	2	F ^3^		[71]
USA	2007	1, 3	neg 5	mild botulism E		[72]
Finland	2011	3	2	B		[74]
Japan	2012	2	2	A		[76]
Italy	2015	1	1	B		[79]
Germany	2018	3	2	A		[81]

^1^ Dates of serum sampling known for 23 of the 28 toxin positive sera, ^2^ From a total of 87 serum samples for which date specimen was obtained, ^3^ *C. baratii* F, ^4^ Sera from 8 patients of the same outbreak, negative detection of BoNT in 6 sera. neg, negative.

**Table 4:** Detection of BoNT in the serum of infants with botulism.

Country	Year	Number of Infant’s Sera	Toxin-Positive Sera	BoNT Type			References
				A	B	E	
USA	1975–1987	67	9	8	1		[90]
Australia	1981	1	0		1 ^1^		[91]
Australia	1982	1	1	1			[92]
Japan	1986–1987	2	2	2			[93]
Italy	1984–2008	12	2		1	1 ^2^	[94,95]
UK	1994–2007	4	4	2	2		[96]
Denmark	1997	1	1	1			[97]
UK	2001	1	0		1 ^1^		[98]
Finland	2002	1	0		1 ^1^		[99]
USA	2007	1	1			1	[100]
Denmark	2008	1	1 ^3^				[101]
Portugal	2009	1	0		1 ^1^		[102]
Finland	2010	1	1	1			[103]
France	2004–2016	16	9	5	4		[48,49,104,105,106,107,108,109].
Denmark	2013	1	1	1			[110]
Argentina	2014	1	1	1			[111]
Total		112	31				

^1^*C. botulinum* B in feces, ^2^ *C. butyricum* E in feces, ^3^ Botulinum toxin type not identified.

**Table 5:** Detection of botulinum toxin in sera of infant botulism according to the time between the symptom onset and serum sampling.

Country	Year	Patient Age at the Onset of Symptoms m, months; d, days	Days from Symptom Onset to Serum Sampling	BoNT in Serum	*C. botulinum* in Feces	References
Australia	1981	4 m	4	neg	*C. botulinum* B	[91]
Australia	1982	7 d	3	A	*C. botulinum* A	[92]
			10	A	*C. botulinum* A	
			16	neg	*C. botulinum* A	
Japan	1986	79 d	22	neg	*C. botulinum* A	[93]
	1987	40 d	7	A	*C. botulinum* A	
			24	A	*C. botulinum* A	
			26	A	*C. botulinum* A	
			27	A	*C. botulinum* A	
			46	neg	*C. botulinum* A	
UK	2007	8 m	15	A	*C. botulinum* A	[113]
UK	2001	5 m	10	neg	*C. botulinum* B	[98]
USA	2007	9 d	5	neg	*C. botulinum* E	[100]
Denmark	2008	4.5 m	14	BoNT ^1^		[101]
Finland	2010	3 m	3	A	*C. botulinum* A	[103]
			15	neg	*C. botulinum* A	
Denmark	2013	5 m	4	A	*C. botulinum* A	[110]
France	2013	2 m	28	neg	*C. botulinum* A	[107]
			48	neg	*C. botulinum* A	
			63	neg	*C. botulinum* A	
Argentina	2014	4 m	3	A	*C. botulinum* A	[111]

^1^ Botulinum toxin type not identified, neg, negative.

**Table 6:** Detection of BoNT in the serum of patients with botulism by intestinal colonization.

Country	Year	Patient Age	Days from Symptom Onset to Serum Sampling	BoNT in Serum	BoNT in Feces	Feces	References
USA	1978–1985	elderly	22	nd	B	*C. botulinum* B	[116]
			30	B	neg	*C. botulinum* B	
			37	neg	nd	nd	
		33	2	A	nd	*C. botulinum* A	
		27	47	B	neg	*C. botulinum* B	
		37	14	A	A	*C. botulinum* A	
			21	neg	neg	*C. botulinum* A	
USA	1985	37	11	A	A	*C. botulinum* A	[117]
			13	A	nd	*C. botulinum* A	
USA	1988	67	3	A	A	nd	[115]
			5	A	A	*C. botulinum* A	
			~20	neg	nd	*C. botulinum* A	
Italy	1994	9	5	neg	nd	*C. butyricum* E	[118]
Italy	1997	56	30	A	neg	*C. botulinum* A	[95]
Japan	2003	12	2	A	A	*C. botulinum* Ab	[119]
Canada	2007	50	13	A	nd	*C. botulinum* A	[120]
France	2011	10		Ind ^1^	nd	*C. butyricum* E	[49]
USA	2017	43	12	A	nd	*C. botulinum* A	[121]
USA	2017	elderly	16	A ^1^	A	nd	[122]
USA	2017	27	78	neg	A	*C. botulinum* A	[123]
USA	2018	66	5	A	A	nd	[124]

^1^ Toxin type undetermined. ^2^ Detection of BoNT/A negative by mouse bioassay and positive by MALDI-TOF MS. nd, not done; neg, negative.

**Table 7:** Detection of BoNT in the serum of patients with wound botulism.

Country	Year	Number of Patient’s Sera	Toxin-Positive Sera	BoNT Type				References
				A	B	E	ind	
California	1951–1998	122	114	84%	12%		4%	[126]
California	1993–2006	37	22	20	2			[127]
California	2005–2007	73	50					[128]
Italy	1986–2015	6	5		5			[80]
UK	2000–2004	88	33	~90%	~10%			[125,129]
Germany	2005	4	2		2			[130]
Sweden	2006	1	0			1 ^1^		[131]
France	2008	1	1		1			[47]
USA	2011	1	1	1				[132]
Germany	2016	1	1		1			[133]
USA	2017	1	1	1 ^2^				[134]
Total		335	230					

^1^*C. botulinum* E detected in the wound by real time PCR, ^2^ *C. subterminale* identified in wound culture.

**Table 8:** Detection of botulinum toxin in sera of patients with neuropathic symptoms mimicking botulism.

Country	Year	Number of Patient’s Sera	Toxin Positive Sera	Additional Investigation	Symptoms	References
The Netherlands	1988–1992	5	4 ^1^		GBS	[145]
The Netherlands	1994	6	1 ^1^	CSF protein: 125 mg/dL, electromyography	GBS	[144]
USA	1998	1	BoNT/B	CSF protein: 42 mg/dL, electrodiagnostic	possible concurrent botulism and MFS	[146]
Germany	2004	1	neg	strongly elevated IgG/IgM anti-GQ1b	MFS mimicking botulism	[159]
France	2007–2009	38	24 ^1^		GBS/MFS	[47] and unpublished data
USA	2015	1	BoNT/A	strongly elevated Ig anti-GQ1b CSF protein: 56 mg/dL negative detection of BoNT and *C. botulinum* in feces	MFS	[147]
USA	2017	1	neg	strongly elevated IgG/IgM anti-GQ1b BoNT in recently ingested canned food	possible concurrent MFS and food-borne botulism	[160]
Switzerland	2017	1	neg	elevated IgG anti-GM1, -GD1a, -GD1b	atypical GBS/MFS mimicking botulism	[136]

^1^ Toxicity in sera not neutralized by specific anti-botulinum toxin type antibodies. GBS, Guillain Barré syndrome; MFS, Miller-Fisher syndrome; neg, negative.

**Table 9:** In vitro methods of BoNT detection in human serum.

BoNT Type	Method	Serum Samples	Sensitivity	References
A	Assay with a large immunosorbent surface area (ALISSA)	spiked human serum	Attomolar (4 × 10^−5^ MLD/mL) ^a^	[168,169]
A, B, C, D, E, F	Endopeptidase assay and monoclonal neoepitope antibodies	spiked human serum	A: 0.211 MLD50/mL B: 0.147 MLD50/mL C: 2.056 MLD50/mL D: 0.18 MLD50/mL E: 0.84 MLD50/mL F: 0.005 MLD50/mL	[170]
A, B, E, F	Endopep-MS	spiked human serum	A: 20 MLD/mL B, F: 1 MLD/mL E: 0.2 MLD/mL	[171]
A, B, E, F	Endopep-MALDI-TOF-MS	spiked human serum	A, B, F: 0.1 MLD/mL E: 0.02 MLD/mL	[172,173,174]
A, B, F	Endopeptidase assay with fluorogenic substrates	spiked human serum	A, F: 1 pM (A: 40 MLD/mL F: 3 MLD/mL) ^a^ B: 10 pM (180 MLD/mL) ^a^	[175,176]
A	Endopep-MS	spiked human serum	1 MLD/mL	[177,178]
A, B	Endopep-MS	spiked macaque serum	1 MLD/mL	[179]
A, B, D/C, E, F	Endopep-MS	spiked human, chicken sera	A, B, F: 1 MLD/mL D/C: 2 MLD/mL E: 10 MLD/mL C: 100 MLD/mL	[180]
C	Endopep-MS	spiked human serum	0.5–1 MLD/mL	[181]
A	Microfluidic double sandwich immunoassay	spiked human serum	30 pg/mL (8 MLD/mL) ^a^	[182]
A	Functional dual coating assay (BoNT immunocapture and endopeptidase assay with detection by neoepitope antibodies)	clinical serum samples	1 MLD/mL	[183,184]
A	Endopeptidase assay and detection with neoepitope antibodies and surface plasmon resonance	clinical serum samples	<1 MLD/mL negative in ELS and CIDP patient’s sera	[167]
A	Endopep-MS	clinical serum samples	~1 MLD/mL	[185]
B	Endopeptidase assay and detection with neoepitope antibodies and surface plasmon resonance	clinical serum samples	0.1–0.01 MLD/mL negative in ELS and GBS patient’s sera	[166]

Endopep-MS, endopeptidase assay and mass spectrometry; ELS, Eaton-Lambert syndrome; CIDP, chronic inflammatory demyelinating neuropathy; GBS, Guillain-Barré syndrome. ^a^ The equivalence in MLD/mL has been determined according to [186].
